# Supplementary material for: Identification of photoperiod-induced specific miRNAs in the adrenal glands of Sunite sheep (Ovis aries)
Source: Front Vet Sci. 2022 Jul 22;9:888207. doi: 10.3389/fvets.2022.888207 (PMC9354845; doi:10.3389/fvets.2022.888207)
Supplement: Supplementary file 1 [file Data_Sheet_1.ZIP › Supplementary file/miRDeep2 file.zip!/miRDeep2 file/Know_Structure/SPLP42A3/oar-let-7c.pdf]

miRBase precursor	: oar-let-7c
Total read count	: 110425
oar-let-7c read count	: 110374
remaining reads	: 51

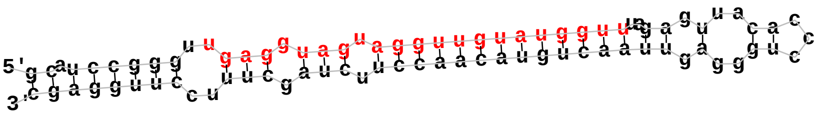

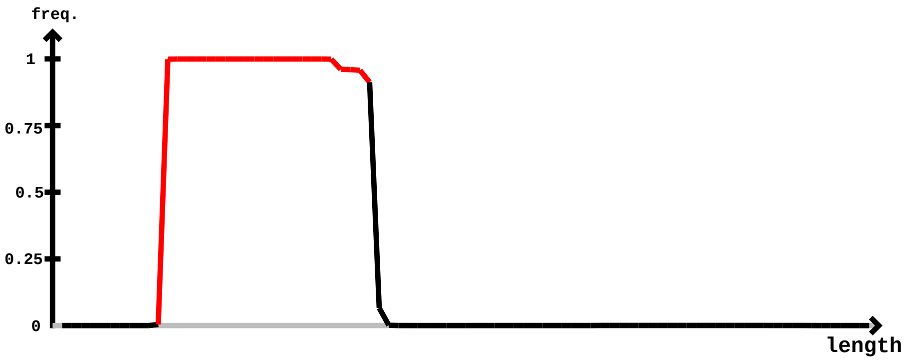


```
                oar-let-7c
5'- gcauccggguugagguaguagguuguaugguuuagaguuacacccugggaguuaacuguacaaccuucuagcuuuccuuggagc   -3'    exp
    ((.((((((..(((.(((.(((((((((((((..((.(..((...))..).))))))))))))))).))).)))..))))))))   reads      mm        sample
    ........guugagguaguagguuguauggu.....................................................   1          0         seq
    .........uugagguaguagguugua.........................................................   1          0         seq
    .........uugagguaguagguuguaug.......................................................   2          0         seq
    .........uugagguaguagguuguaugg......................................................   8          0         seq
    .........uugagguaguagguuguauggu.....................................................   76         0         seq
    .........uugagguaguagguuguauAgu.....................................................   2          1         seq
    .........uugagguaguagguuguaugguu....................................................   334        0         seq
    .........uugagguaguagguuguauAguu....................................................   7          1         seq
    .........uugagguaguagguuguaugguuu...................................................   17         0         seq
    ..........ugagguaguagguugua.........................................................   54         0         seq
    ..........ugagguaguaAguugua.........................................................   1          1         seq
    ..........ugagguagGagguugua.........................................................   16         1         seq
    ..........ugagguaguagguuguau........................................................   3808       0         seq
    ..........ugagguaguaAguuguau........................................................   12         1         seq
    ..........ugagguUguagguuguau........................................................   4          1         seq
    ..........ugagguagGagguuguau........................................................   337        1         seq
    ..........ugaAguaguagguuguau........................................................   30         1         seq
    ..........ugagguaguagguuUuau........................................................   16         1         seq
    ..........ugagguaguagguuguaug.......................................................   96         0         seq
    ..........ugagguaguagguuUuaug.......................................................   1          1         seq
    ..........ugagguaguagguuguaugg......................................................   297        0         seq
    ..........ugagguaguagguuguauggu.....................................................   4858       0         seq
    ..........ugagguaguagguuguaugguu....................................................   93074      0         seq
    ..........ugagguaguagguuguaugguuu...................................................   7124       0         seq
    ..........ugagguaguagguuguauAguuua..................................................   92         1         seq
    ..........ugagguaguagguuguaugguuua..................................................   25         0         seq
    ..........ugagguaguagguuguaugguuuaga...............................................   2          0         seq
    ...........gagguaguagguuguauggu.....................................................   7          0         seq
    ...........gagguaguagguuguaugguu....................................................   60         0         seq
    ...........gagguaguagguuguaugguuu...................................................   9          0         seq
    ............agguaguagguuguaugguu....................................................   1          0         seq
    .............gguaguagguuguaugguu....................................................   1          0         seq
    .................guagguuguaugguuuU..................................................   1          1         seq
    ................................uagaguuacacccugggagu................................   1          0         seq
    ................................uagaguuacacccugggaguu...............................   3          0         seq
```

oar-let-7c

```
gcauccgggu**ugagguaguagguuguaugguu**uagaguuacacccugggaguuaacuguacaaccuucuagcuuuccuuggagc
.........................................acccugggaguuaacuguacaacc...................    1    0    seq
..........................................cccugggaguuaacuguacaacc...................    1    0    seq
..............................................gggaguuaacuguacaaccuucua..............    1    0    seq
................................................gaguuaacuguacaaccuucuagcu...........    1    0    seq
.................................................aguuaacuguacaaccuucu...............    1    0    seq
.................................................aguuaacuguacaaccuucuagcu...........    4    0    seq
.................................................aguuaacuguacaaccuucuagcuuucc.......    1    0    seq
....................................................uaacuguacaaccuucuagcu...........    1    0    seq
.......................................................cuguacaaccuucuagcuuu.........    2    0    seq
.......................................................cuguacaaccuucuagcuuuc........    4    0    seq
.......................................................cuguacaaccuucuagcuuucc.......    20   0    seq
.......................................................cuguacaaccuucuagcuuuccu......    9    0    seq
........................................................uguacaaccuucuagcuuuccu......    1    0    seq
```
